# Careful CO Addition Enhances Autotrophic d‐Lactate Formation With Engineered *Acetobacterium woodii*

**DOI:** 10.1002/elsc.70072

**Published:** 2026-02-17

**Authors:** Anna Stock, Inka Sotzeck, Kira Baur, Frank Bengelsdorf, Dirk Weuster‐Botz

**Affiliations:** ^1^ Chair of Biochemical Engineering Technical University of Munich Garching Germany; ^2^ Institute of Molecular Biology and Biotechnology of Prokaryotes University of Ulm Ulm Germany

**Keywords:** *Acetobacterium woodii*, CO conversion, d‐lactate production, metabolic engineering, syngas fermentation

## Abstract

The acetogen *A. woodii* efficiently converts CO_2_ and H_2_ to acetate. Metabolic engineering enabled the autotrophic production of non‐native products, for example, d‐lactate from CO_2_ by overexpression of d‐lactate dehydrogenase from *Leuconostoc mesenteroides* and knockout of the native lactate dehydrogenase. During gas fermentation with acetogens, the addition of CO leads to increased provision of reducing equivalents, and thus increased biomass formation. However, literature data reveal that already small CO partial pressures in the gas phase inhibit the autotrophic growth of *A. woodii*. This study aims to investigate adding 0.6%–6.0% CO to batch‐operated stirred tank bioreactors with continuous gassing to study autotrophic growth and product formation with the d‐lactate producing *A. woodii* mutant. No growth and product formation were observed with 6% CO. Surprisingly, cell growth and metabolic product concentrations are non‐linearly dependent on lower CO concentrations in the inlet gas phase. Highest biomass concentrations were observed with 3% CO (3.24 g L^−1^, 89% improvement compared to the reference process without CO addition), and the highest d‐lactate accumulation was achieved with 0.8% CO (6.2 g L^−1^ d‐lactate, 189% improvement compared to the reference without CO) after a prolonged lag phase. In conclusion, CO‐sensitive *A. woodii* cells need tight control of CO in syngas to affect autotrophic product selectivities.

## 1 Introduction

The reuse of CO_2_ (carbon capture and utilization, CCU) is an essential part of the UN Sustainable Development Goals. Fossil carbon‐intensive industries need to find ways to reuse CO_2_ gas emissions in a cost‐effective manner, if no renewable resources are available. Converting CO_2_ from exhaust gases with microorganisms into water‐soluble, higher‐value products may be an option. Anaerobic acetogenic bacteria are able to grow with H_2_ + CO_2_ and/or CO as carbon and energy sources (syngas fermentation). Typical products are acetate, ethanol, butyrate, 1‐butanol, and other bio‐commodities [1].

**Practical Applications**

The ability to convert CO_2_ and H_2_ efficiently into d‐lactate using engineered *Acetobacterium woodii* has significant industrial implications. D‐lactate is a key precursor for biodegradable plastics, such as polylactic acid (PLA), and serves as an important platform chemical for pharmaceuticals and specialty chemicals. This study demonstrates that controlled CO addition can enhance biomass formation and product selectivities, depending on the amount added. Therefore, it provides a potential strategy to optimize gas fermentation processes. These findings offer insights into process control strategies for improving microbial syngas conversion, making biological CO_2_ utilization more viable for large‐scale biotechnological applications. Additionally, the nonlinear effects of CO on the growth and product selectivity of engineered *A. woodii* highlight the importance of gas composition control in industrial‐scale fermentations. Ultimately, these results contribute to advancing sustainable bioproduction processes that integrate carbon capture with value‐added chemical synthesis, potentially reducing dependence on petrochemical feedstocks in a circular bioeconomy.

Well‐studied anaerobic bacteria for syngas fermentation include acetogens like *Clostridium autoethanogenum*, *Clostridium carboxidivorans*, *Thermoanaerobacter kivui*, *Moorella thermoacetica*, or *Acetobacterium woodii* [2, 3, 4, 5, 6, 7, 8, 9, 10, 11]. Various microorganisms can thus be employed depending on the specific demands regarding product spectrum, gas mixture, and cultivation conditions. Additionally, even though genetic engineering was initially challenging some years ago due to the initial lack of molecular tools, more and more acetogens are becoming genetically accessible [12, 13, 14, 15, 16, 17], and adaptive laboratory evolution approaches are gaining ground for strain improvements [18]. Moreover, successful immobilization approaches have been demonstrated for example, for *A. woodii, M. thermoacetica*, and *Clostridium ljungdahlii*, indicating their potential for continuous or intensified bioprocesses [19, 20]. Among these, *A. woodii* stands out as a widely used model organism—not only due to its well‐characterized bioenergetics, but also because it typically achieves the highest acetate concentrations reported among acetogens. Under optimized conditions, it can convert H_2_ and CO_2_ to up to 59.3 g L^−^^1^ acetate within 3.1 days at elevated pressure in a batch operated stirred tank bioreactor [21, 22]. In addition, genetic engineering of *A. woodii* has enabled both improved acetate production [23] and expansion of its natural product spectrum. While various recombinant products such as acetone [24], hexanoate [25], isopropanol [26], poly‐3‐hydroxybutyrate [27], and d‐lactate [28] have been successfully produced with *A. woodii*, achieving native d‐lactate production remains more complex. Although the organism's endogenous lactate dehydrogenase/electron transferring flavoprotein (LDH/Etf) complex is theoretically reversible [29, 30], earlier studies primarily observed lactate utilization, and lactate production was only possible following hydrogenase deletions [31, 32]. Only one study observed autotrophic lactate production by *A. woodii* wildtype (0.24 g L^−^^1^ of d‐lactate within the first 8 h of a batch cultivation). This, however, was re‐metabolized in the subsequent hours of the cultivation process [4]. Mook et al. enabled recombinant d‐lactate production with *A. woodii* by plasmid‐based overexpression of the d‐lactate dehydrogenase (LDHD) gene (*ldhD*) from *Leuconostoc mesenteroides* in a strain designated as *A. woodii* [P*_bgaL__ldhD_*NFP], controlled by the lactose‐inducible promotor P*_bgaL_* [33]. Simultaneously, the genes encoding the gene products responsible for lactate consumption (*lctBCD*) were knocked out [28, 29]. This enabled the production of high amounts of d‐lactate from CO_2_ and H_2_ in a continuously gassed stirred tank bioreactor with high yeast extract supplementation (6 g L^−1^). Even though acetate remained the main product, 8.1 g L^−1^ d‐lactate was produced resulting in a lactate to acetate ratio of 0.50 g g^−1^ within 82.5 h in the batch process [4]. In this strain, d‐lactate formation was enabled with a lactose‐inducible promoter. Due to high d‐lactate formation, this strain reached lower maximum OD_600_ of 2.07 compared to the *A. woodii* wild type (OD_600_ = 2.70) under similar conditions [34] during gas fermentation with H_2_ and CO_2_. Even worse, growth of the engineered *A. woodii* was nearly stalling after induction with lactose. Given that the engineered strain was cultivated with substantially higher yeast extract concentrations, the discrepancy in biomass formation may even be underestimated.

**FIGURE 1:**
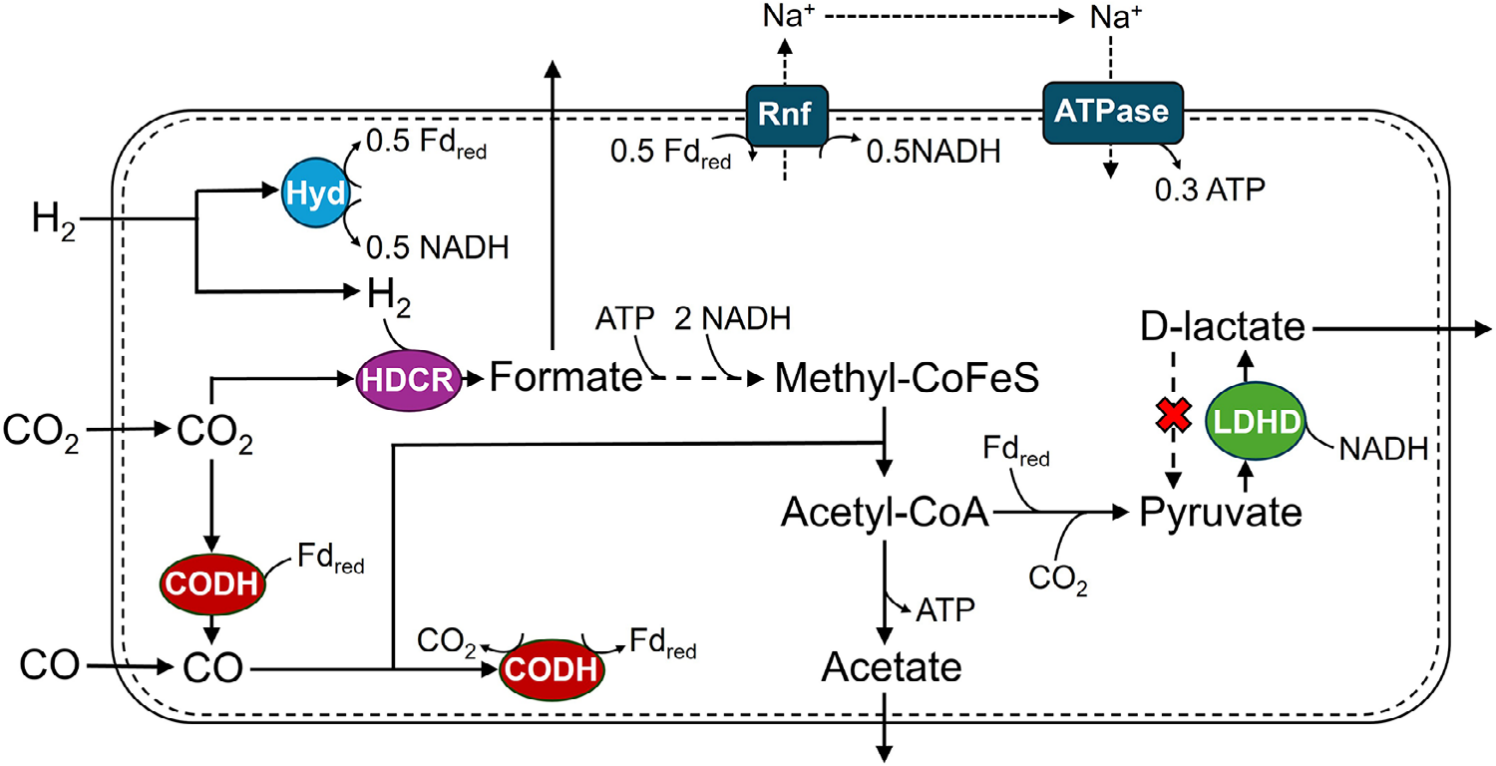
Simplified scheme of the autotrophic product formation and energy metabolism of the engineered D‐lactate producing *A. woodii*. Adapted from Bertsch et al. [36] and Mook et al. [28]. The simplified WLP reactions are stoichiometrically balanced for the formation of 1 mol acetyl‐CoA, whereas the energy conservation and respiratory modules are depicted with balances specific to the respective enzymes rather than for the overall pathway.

To eliminate the need for external lactose addition and to decrease metabolic stress through lactose induction, the lactate‐inducible promoter P*_lctA_* [30] was implemented, resulting in the self‐inducing d‐lactate producer *A. woodii* Δ*pyrE* Δ*lctBCD* [pMTL83251_PlctA_NFP]. The promoter P*_lctA_* originating from *A. woodii* DSM 1030 natively controls its gene *lctA* (Awo_c08700) encoding the transcriptional regulator LctA. LctA binds to the specific bindings site TGGT(CTG)ACCA which is located in the intergenic region of *lctA* and the *lctBCDEF* operon (Awo_c08710‐ Awo_c08760) and prevents its transcription. The repression of the *lctBCDEF* operon is repealed if lactate binds LctA, which leads to the dissociation of LctA from its binding site and subsequent expression of the entire operon [30]. Small quantities of lactate are sufficient to remove LctA regulator since the regulation of the *lctBCDEF* operon is slightly leaky. Thus, the P*_lctA_* promoter was most suitable to establish a self‐inducing and lactate producing *A. woodii* strain. Figure 1 shows relevant aspects of the Wood‐Ljungdahl pathway (WLP) for CO_2_/CO fixation [35], the energy metabolism and the recombinant D‐lactate formation of the engineered *A. woodii* d‐lactate producer.

During gas fermentation with acetogens, the addition of CO leads to the increased provision of reducing equivalents compared to cultivations with H_2_ as sole electron donor. The underlying reason for this is the lower redox potential of the CO/CO_2_ couple (−520 mV) compared to the H_2_/H^+^ couple (−414 mV), enabling more efficient electron donation for cellular redox and energy metabolism. This results in higher maximum cell dry weight concentrations compared to cultivations without CO [37]. The reason for this is the CO dehydrogenase (CODH) that catalyzes the reversible oxidation of CO to CO_2_, while efficiently providing reducing equivalents in the form of reduced ferredoxin (Fd_red_). Fd_red_ generated via CODH may drive the ferredoxin:NAD^+^ oxidoreductase (Rnf) complex, increasing ATP formation. The additional ATP can be redirected toward anabolic processes, which may reduce the cellular demand for catabolic acetate production [36]. Studies with various acetogens demonstrated, that altering the CO concentration can therefore greatly influence biomass formation, product distributions and yields [8, 38, 39]. For example, higher CO concentrations promoted the production of the more reduced ethanol over acetate with *M. thermoacetica* [3].

If CO is absent, *A. woodii* requires Fd_red_ to reduce CO_2_ to CO in the carbonyl branch of the WLP. With H_2_ as the sole electron donor, reducing equivalents are provided by the electron‐bifurcating hydrogenase (HydABC) that catalyzes the reversible oxidation of H_2_, leading to the exergonic production of 0.5 mol NADH and endergonic production 0.5 mol Fd_red_ per mole H_2_ [36, 40]. During cultivations with CO‐containing gas mixtures, the bypassing of the hydrogenase‐dependent H_2_ oxidation combined with the more direct generation of Fd_red_ via CODH, may enable more efficient ATP formation via the Rnf complex and ATP synthase compared to H_2_ + CO_2_ alone. This could result in increased biosynthesis of biomass, accompanied by a reduction in acetate production.

However, the growth of many acetogens is known to be inhibited by CO. The reason for this is its toxicity to [FeFe] hydrogenases, which are critical for the metabolism of some acetogens [36], and the inability to sufficiently redirect electrons toward alcohol production [41]. For the mainly acid‐producing *A. woodii*, it has been shown that CO affects both the HydABC and the H_2_ dependent CO_2_ reductase (HDCR) [42]. Experiments in anaerobic bottles revealed, that already an initial concentration of 5% CO in the gas phase inhibited autotrophic biomass formation. After the addition of formate (to skip the HDCR‐catalyzed CO_2_ reduction in the methyl branch of the WLP, see Figure 1), biomass accumulation was restored and anaerobic growth with CO concentrations of up to 25% (v/v) was enabled. [34, 37] Even though *A. woodii* can be adapted to grow with only CO in the gas phase through the deletion of the [FeFe] hydrogenases HydA2 and HydBA, the adaptation to do so takes a long time [42].

The impact of CO addition on growth and d‐lactate production by engineered *A. woodii* has not been studied prior to this work. The rationale for adding CO to the gas phase with this strain was to improve energy availability for biomass formation and simultaneously support redox balance for d‐lactate production. The additionaly expressed d‐lactate dehydrogenas reduces pyruvate to d‐lactate using NADH as an electron donor, thereby increasing the cellular NADH demand. While H_2_ oxidation via HydABC provides both NADH and Fd_red_, CO oxidation via CODH yields only Fd_red_, but at high catalytic efficiency. This Fd_red_ can drive the Rnf complex, enabling ATP synthesis. The additional ATP generated through this route may reduce the need to oxidize NADH for energy generation, allowing more NADH to be channeled toward d‐lactate formation.

In the former *A. woodii* strain with the lactose‐inducible LDHD expression [4], d‐lactate production was predominantly coupled to induction via lactose. In contrast, in the self‐inducing strain, LDHD expression is primarily coupled to NADH availability and therefore directly dependent on the redox state of the cell. This makes this strain responsive to redox state changes already from the beginning of the cultivation.

To avoid inhibition of hydrogenase activity, only low CO concentrations were applied in this study. Rather than targeting conventional syngas utilization, our objective is to integrate CO_2_/H_2_ fermentation with small, on‐site sources of, for example, electro‐catalytically produced CO. Such systems typically deliver CO in the low‐percent range, making the tested window of 0.6%–6 % (v/v) CO technologically realistic. [43] Therefore, we systematically tested different CO concentrations below 6% CO keeping H_2_ and CO_2_ constant to determine the tolerable level and its effect on growth and d‐lactate production of the newly constructed, D‐lactate producing *A. woodii*. Fully controlled, continuously gassed, stirred tank bioreactors will be applied for the batch operated gas fermentations to ensure sufficient gas–liquid mass transfer at well‐defined reaction conditions.

## 2 Materials and Methods

### 2.1 Organism and Strain Engineering

*A. woodii* ∆*pyrE* ∆*lctBCD* was used as a parental strain [44] to construct the new lactate producing *A. woodii* mutant used in this study. Therefore, a new plasmid termed pMTL83251_P*_lctA_* _NFP was constructed by exchanging the promoter region in the plasmid pMTL83251_P*_bgaL_* _NFP constructed by Mook et al. [28]. This plasmid DNA was linearized by using the restriction enzymes *Not*I and *Bam*HI and the promoter P*_bgaL_* fragment cut out by creating overlaps to introduce the promoter P*_lctA_*. The fragment P*_lctA_* was amplified with the primers PlctA_fwd (attcgagctcggtacccgggtcaggacttatcaagtttaagt) and PlctA_rev (ccatggatccactcgccctccattaaattaattaaag). The plasmid pMTL83251_P*_lctA_* _NFP was assembled using the NEBuilder HiFi DNA Assembly Kit (New England Biolabs, Ipswich, Ma, USA) and replicated in chemocompetent *E. coli* DH5α [45]. Respective plasmid DNA was isolated from *E. coli* cells, sequenced and verified. Thus, expression of the LDHD encoded on the plasmid pMTL83251_P*_lctA_* _NFP is controlled by the promoter P_lctA_. The details of the expressed fusion gene *NFP_awo_opt* (GenBank‐Nr. OL439953) were already described by Mook et al. 2022. Briefly, it consists of the codon optimized variant of the *ldhD* gene from *L. mesenteroides* (GenBank‐Nr. OL439952) connected with a single GGGGS linker with a codon optimized variant of *feg2* (GenBank‐Nr. OL439951), the sequence for FAST2, which is a fluorescent protein tag [28].

Electro‐competent cells of *A. woodii ∆lctBCD ∆pyrE* were prepared as described previously and transformed with the plasmid pMTL83251_P*_lctA_* _NFP [24]. The transformed *A. woodii* cells were verified by isolating and sequencing the plasmid DNA.

### 2.2 Media

*A. woodii* cells were cultivated in a phosphate buffered medium as previously described [29] with an increased yeast extract concentration of 3.0 g L^− 1^ and additional 20 mg L^−1^ uracil due to the auxotrophy of the engineered *A. woodii*. Plasmid stability was ensured by the addition of 5 mg L^− 1^ clarithromycin. For the precultures, the phosphate buffer concentrations were increased to 8.44 g L^−1^ K_2_HPO_4_ and 1.76 g L^−1^ KH_2_PO_4_ [29]. Media compositions are listed in the supplementary material (Tables S1–S3). Preparation of the medium followed anaerobic preparation protocols [46, 47].

### 2.3 Precultures

Precultures were grown in anaerobic 500 mL flasks containing 100 mL medium, incubated at 30°C and 110 rpm in an incubator (WIS‐20, Witeg, Wertheim, Germany). The headspaces of the anaerobic flasks were gassed with 70% (v/v) H_2_, and 30% (v/v) CO_2_ if no CO was added in the following stirred tank bioreactor cultivations. For bioreactor cultivations including CO, precultures were prepared with a gas mixture consisting of 70% (v/v) H_2_, 27% (v/v) CO_2_, and 3% (v/v) CO. An absolute pressure of 2.0 bar was applied in the anaerobic flasks. For preparing the cell stock solutions, cells growing anaerobically with 10 g L^−1^ fructose were supplied with 10% DMSO in the exponential phase and subsequently stored at −80°C.

All precultures were prepared with two stages. In the first stage, 2.5 mL of the thawed cell stock solutions were grown mixotrophically with 10 g L^−1^ fructose for ∼60 h. For the second stage, three times 15 mL of the cell suspensions were transferred into new anaerobic flasks and grown autotrophically for 24 h. To inoculate the stirred tank bioreactor, these cells were centrifuged (20 min, 3620 g (rcf), Rotica 50 RS, Hettich GmbH & Co. KG, Tuttlingen, Germany) and the emerging pellet was suspended in 10 mL anaerobic phosphate‐buffered saline (PBS, 0.20 g L^−1^ KCl, 0.24 g L^−1^ KH_2_PO_4_, 8.00 g L^−1^ NaCl and 1.44 g L^−1^ Na_2_HPO_4_). All bioreactor cultivations were inoculated through a septum (diameter 12 mm, Infors AG, Germany) using a sterile, single‐use syringe (BD Discardit II; Becton Dickinson, Franklin Lakes, NJ, USA) and a sterile needle (Sterican 0.9 × 70 mm, B. Braun, Melsungen, Germany).

### 2.4 Stirred Tank Bioreactor Cultivations

All batch cultivations were conducted in a fully controlled, continuously gassed, stirred tank reactor with a total volume of 3.7 L, and a working volume of 2.0 L (KLF, Bioengineering, Wald, Switzerland). The cell suspension was stirred at 1000 rpm using two Rushton turbines and four baffles enabling a volumetric power input of 6.06 kW L^−1^ to ensure high H_2_ (and CO) gas–liquid transfer rates. The pH and the redox potential were continuously monitored using autoclavable sensors (405‐DPAS‐SC‐K8s/120, Mettler Toledo, Giesen, Germany, and Pt4805‐DPAS‐SC‐K8S/120, Mettler Toledo, Germany, respectively). The pH was kept constant at pH 7.0 using 6 M KOH and the temperature was controlled at 30°C using a heating and a cooling rod.

During batch cultivations, the reactor was continuously gassed with a mixture of H_2_, CO_2_, CO or N_2_ at a gas flow rate of 0.25 vvm through a sintered sparger at the bottom of the stirred tank bioreactor. Independent mass flow controllers enabled defined addition of the specific gas components in the desired ratios (F‐201CV‐500 RGD‐33‐V, Bronkhorst High‐Tech B.V., Ruurlo, Netherlands). The stirred tank bioreactor was operated at an absolute pressure of 1.0 bar. To avoid media loss through evaporation, the off‐gas pipe was cooled to 2°C using a cooling jacket.

The stirred tank bioreactor was sterilized in situ at 121°C for 20 min with the medium without clarithromycin, l‐cysteine hydrochloride and vitamins. Starting during cooling, the medium was gassed with 0.167 vvm N_2_ for at least 16 h to strip the O_2_ and to ensure anaerobic conditions. Subsequently, the synthetic syngas mixture was sparged through the bioreactor for at least 3 h prior to cultivation start. After the medium in the bioreactor had cooled down to temperatures below 40°C, clarithromycin, L‐cysteine hydrochloride, and vitamins were added using sterile single‐use syringes (BD Discardit II, Becton Dickinson, Franklin Lakes, NJ, USA) with microfiltration membrane filters (pore size 0.2 µm, VWR, Radnor, PA, USA) and sterile needles (Sterican 0.9 × 70 mm, B. Braun, Melsungen, Germany). All cultivations were inoculated at cell dry weight concentrations between 0.024–0.06 g L^−1^. For sampling and adding heat‐sensitive chemicals, sterile needles (Sterican 0.9 × 70 mm, B. Braun, Melsungen, Germany) and septums (diameter 12 mm, Infors AG, Germany) installed on the jacket of the stirred‐tank reactor were used.

### 2.5 Analytical Methods

For sampling, 2–5 mL of cell suspension was withdrawn through a septum (diameter 12 mm, Infors AG, Germany) from the bioreactor using a single‐use syringe with a sterile needle.

For the estimation of the cell dry weight (CDW) concentrations, the optical density OD_600_ was measured in technical triplicates at 600 nm in a UV–vis spectrophotometer (Genesys 10S UV–vis, Thermo Scientific, Neuss, Germany). To estimate the biomass concentration, a linear correlation factor of 0.49 g L^−1^ OD_600_^−1^ was used, which was identified initially based on cell dry weight measurements: 6×50 mL *A. woodii* cell broth was anaerobically transferred to dried and pre‐weighted 50 mL Falcon tubes (Cellstar Tubes 50 mL, Greiner Bio‐one GmbH, Germany), centrifuged (20 min, 3620 g (rcf), Rotica 50 RS, Hettich GmbH & Co. KG, Tuttlingen, Germany), washed with anaerobic PBS and centrifuged again. The supernatant was discarded, and cells were dried in a drying chamber (UN260, Memmert, Schwabach, Germany) at 80°C for 72 h.

Product concentrations were determined via high‐performance liquid chromatography (LC‐2030C, Shimadzu, Kyoto, Japan) with a cation exchange column (Aminex HPX‐87H, Bio‐Rad, Munich, Germany) using a refractive index detector (RID‐20A, Shimadzu, Kyoto, Japan). Separation was conducted using 5 mM H_2_SO_4_ as a solvent at an isocratic, constant flow rate of 0.6 mL min^−1^ and a column temperature of 60°C. Prior to each measurement, cell suspension was filtered (Chromafil RC20/15 MS; Macherey‐Nagel GmbH & Co.KG, Düren, Germany), diluted (with PBS, if necessary) and stored at 4°C.

The volumetric exhaust gas flow rate of the bioreactor was measured online using a mass flow meter (F‐111B‐1K0‐RGD‐33‐E; Bronkhorst High‐Tech B.V., Ruurlo, the Netherlands). Every 10 min, a micro gas chromatograph (µGC, micro‐GC 450, Agilent Technologies, Waldbronn, Germany) analyzed the off‐gas composition with a 1 m Cox HI column (molecular sieve, nitrogen as carrier gas, column temperature 100°C, initial pressure 200 kPa) using a thermal conductivity detector (Agilent Technologies, Waldbronn, Germany). H_2_ and CO_2_ uptake rates were calculated using the measured exhaust gas flow rates and the related gas compositions obtained by the µGC. Due to the low concentrations of CO in the exhaust gas, CO could not be detected by the micro gas chromatograph. Alternatives for the analysis of the solved CO concentrations, like for example by application of a non‐commercially available CO probe [48], or the off‐line application of a myoglobin assay [49] were not available (CO probe) or will not enable the estimation of CO uptake rates with sufficient accuracy. Since the CO uptake by the cells was thus not measurable in the stirred tank bioreactor, carbon balance estimations ignored CO. Nevertheless, the carbon balance of all batch processes with continuous gassing were closed with a carbon recovery of 90%–110% within the estimation error.

## 3 Results and Discussion

### 3.1 Gas Fermentations With and Without CO Addition

**FIGURE 2:**
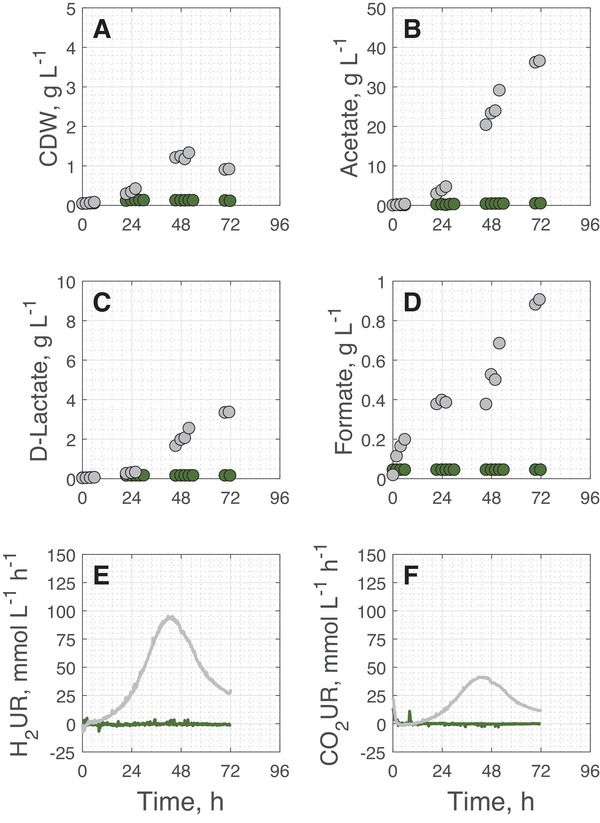
Cell dry weight (CDW) (A), acetate (B), d‐lactate (C), formate (D) concentrations, and H_2_ (E) and CO_2_ uptake rates (F) during gas fermentation with *A. woodii* Δ*pyrE* Δ*lctBCD* [pMTL83251_P*_lctA_* _NFP] in a batch operated stirred tank bioreactor with continuous gassing. Data shown in green represent results with 6% (v/v) CO and data shown in gray without additional CO in the inlet gas (*T* = 30°C; pH  =  7.0; P V^−1^ = 6.06 W L^−1^, *F*_gas_  =  0.08 vvm). We selected solely the representative H_2_ and CO_2_ uptake rates from one of the two independent processes for a more explicit representation.

Initially, two batch processes were carried out in stirred tank bioreactors with continuous gassing (0.08 vvm) with and without the addition of 6% (v/v) CO to the inlet gas. Cell growth, as well as acetate, formate, and d‐lactate formation dynamics of both cultivations are compared in Figure 2. In addition, H_2_ and CO_2_ consumption rates are shown as function of process time. All key process performance data is summarized in Table 1.

**TABLE 1:** Process performance data of autotrophic batch studies with *A. woodii ΔpyrE ΔlctBCD* [pMTL83251_PlctA_NFP]. Data represents maximum concentrations and maximum growth, production and uptake rates measured without and with 6% CO in the inlet gas in fully controlled stirred tank reactor cultivations with continuous gassing at 0.08 vvm. (*T*  =  30°C; pH  =  7.0; P V^−1^ = 6.06 W L^−1^, *F*_gas_  =  0.25 vvm, c_X0_ = 0.05 g L^−1^ CDW).

	H_2_ + CO_2_ and 0% CO	H_2_ + CO_2_ and 6% CO	Change with / without CO
CDW, g L^−1^	1.33	0.14	−89%
µ_max_, h^−1^	0.13	0.01	−92%
c_Acetate_, g L^−1^	36.62	0.55	−99%
Q_Acetate_, g L^−1^ h^−1^	1.17	0.07	−94%
c_Lactate_, g L^−1^	3.38	0.17	−95%
Q_Lactate_, g L^−1^ h^−1^	0.12	0.01	−92%
c_Formate_, g L^−1^	0.91	0.05	−95%
Q_Formate_, g L^−1^ h^−1^	0.09	0	/
c_Acetate_ / c_Lactate_, g g^−1^	10.83	3.24	−70%
H_2_UR, mmol L^−1^ h^−1^	95.34	4.91	−95%
CO_2_UR, mmol L^−1^ h^−1^	41.41	10.99	−73%
H_2_UR / CO_2_UR, mol mol^−1^	2.30	0.45	−80%

Compared to the reference process without CO addition, cells in the batch process with additional 6% CO showed nearly no growth, gas uptake or product formation. During cultivation without CO addition, cell growth started after ∼ 6 h. The maximum measured CDW concentrations were 1.33 g L^−1^ after 50 h (Figure 2A). However, biomass concentrations decreased afterwards. A reason for the decay could be a limitation in uracil, which was initially added to the medium due to the uracil auxotrophy of this *A. woodii* strain. 20 mg L^−1^ uracil could have been too low, even though the yeast extract in the medium may contain significant amounts of uracil.

The maximum acetate (36.6 g L^−^^1^, Figure 2B) and formate concentrations (0.9 g L^−^^1^, Figure 2D) observed during gas fermentation without CO as well as maximum gas uptake rates (94.4 mmol L^−1^ h^−1^ H_2_ and 40.9 mmol L^−1^ h^−1^ CO_2_, Figure 2E,F) are consistent with values reported in other studies of *A. woodii* using continuously gassed stirred tank bioreactors [34]. Additionally, the newly introduced, self‐inducible promoter resulted in the production of 3.4 g L^−1^ d‐lactate without CO addition (Figure 2C).

Previously published results have shown that the addition of low concentrations of CO (5%–7.5%, v/v) in anaerobic bottle experiments can markedly influence acetate formation and H_2_ uptake during autotrophic growth of *A. woodii* [37]. However, in the present study, a substantially stronger inhibitory effect was observed already at 6% (v/v) CO in the continuously gassed stirred tank bioreactor. Due to the improved gas–liquid mass transfer in the stirred tank bioreactor operated at high volumetric power input (> 6 W L^−1^) and with continuous gassing, most probably higher dissolved CO concentrations may lead to stronger inhibition of key metabolic functions. Although *A. woodii* has previously been cultivated in stirred tank bioreactors with CO [34], these studies consistently supplemented formate to stimulate growth, which was not applied here.

### 3.2 Gas Fermentations With 3% CO Addition at Varying Gas Flow Rates

**FIGURE 3:**
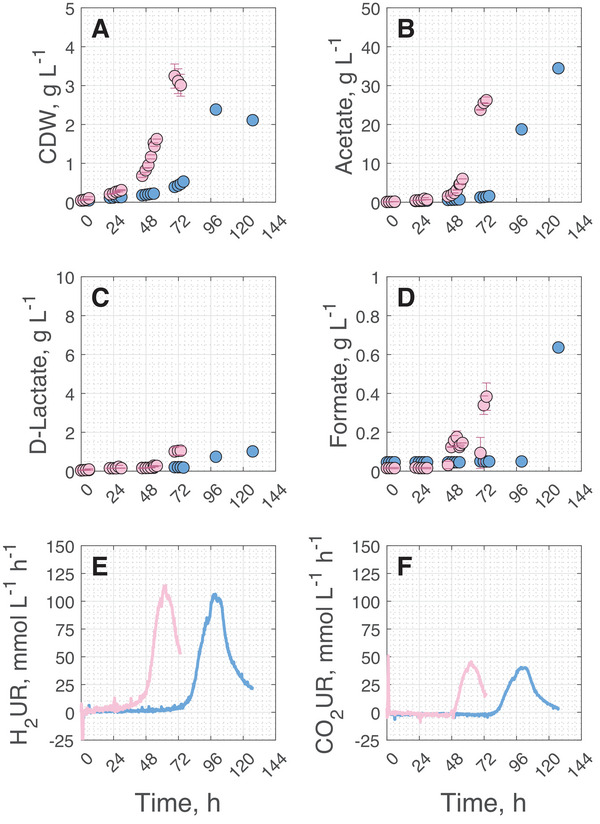
Cell dry weight (CDW) (A), acetate (B), d‐lactate (*C*), formate (D) concentrations, and H_2_ (E) and CO_2_ uptake rates (F) during gas fermentation with *A. woodii* Δ*pyrE* Δ*lctBCD* [pMTL83251_P*_lctA_* _NFP] in batch operated stirred tank bioreactors with continuous gassing of 3% (v/v) CO in the inlet gas. Data shown in rose represent results with a gas flow rate of 0.25 vvm and data shown in light blue represent results with a gas flow rate of 0.08 vvm (*T* = 30°C; pH  =  7.0; P V^−1^ = 6.06 W L^−1^). The error bars during cultivations at 0.25 vvm indicate the minimum and maximum values of two independent batch processes under autotrophic growth conditions. We selected solely the representative H_2_ and CO_2_ uptake rates from one of the two independent processes for a more explicit representation.

To reduce the strong inhibitory effect of CO observed previously, the CO concentration in the inlet gas was lowered to 3% (v/v). Batch cultivations were then performed at 0.08 vvm as before, and 0.25 vvm to vary the gas‐liquid mass transfer rates. The results of the gas fermentation processes with engineered *A. woodii* are shown in Figure 3 and the key process performance data is presented in Table 2.

**TABLE 2:** Process performance data of autotrophic batch studies with *A. woodii ΔpyrE ΔlctBCD* [pMTL83251_PlctA_NFP]. Data represents maximum concentrations and maximum growth, production and uptake rates measured with 3% CO in the inlet gas at 0.08 and 0.25 vvm in fully controlled stirred tank reactor cultivations. (*T*  =  30°C; pH  =  7.0; P V^−1^ = 6.06 W L^−1^, *F*_gas_  =  0.25 vvm).

	0.08 vvm	0.25 vvm	Change 0.25 / 0.08 vvm
CDW, g L^−1^	2.38	3.24	+ 36%
µ_max_, h^−1^	0.08	0.18	+125%
c_Acetate_, g L^−1^	34.46	26.23	−24%
Q_Acetate_, g L^−1^ h^−1^	0.72	1.29	+ 79%
c_Lactate_, g L^−1^	1.02	1.05	+ 3%
Q_Lactate_, g L^−1^ h^−1^	0.02	0.06	+ 200%
c_Formate_, g L^−1^	0.64	0.38	−41%
Q_Formate_, g L^−1^ h^−1^	0.02	0.10	+400%
c_Acetate_ / c_Lactate_, g g^−1^	33.78	24.98	−26%
H_2_UR, mmol L^−1^ h^−1^	106.47	113.98	+ 7%
CO_2_UR, mmol L^−1^ h^−1^	40.54	44.86	+ 11%
H_2_UR / CO_2_UR, mol mol^−1^	2.63	2.54	−3%

Growth of the engineered *A. woodii* strain, gas consumption as well as product formation were observed at 3% (v/v) CO in the inlet gas. In contrast to the reference batch process without CO showing a lag‐phase of 12 h (see Figure 2), prolonged lag‐phases of 48 h, and 72 h were observed with 3% CO at 0.25 vvm, and 0.08 vvm, respectively, indicated by the onset of the H_2_‐uptake rates (Figure 3F). In contrast to cultivations without CO, H_2_ uptake (Figure 3E) was observed prior to CO_2_ uptake at both gas flow rates, most likely due to the presence of CO as carbon source. Additionally, increasing the gas flow rate from 0.08 to 0.25 vvm with 3% CO led to a markedly shorter lag‐phase in the stirred tank bioreactor. The maximum CDW concentrations were increased at 0.25 vvm by 36% compared to 0.08 vvm (Figure 3A). Final product concentrations cannot be compared because both gas fermentation processes were finished at varying final gas consumption rates. Compared to the reference process without CO addition (Figure 2A), maximum CDW concentrations were substantially increased with 3% CO (3.24 g L^−1^ at 0.25 vvm, and 2.48 g L^−1^ at 0.08 vvm compared to 1.33 g L^−1^ without CO), but d‐lactate concentrations are clearly reduced after 72 h.

Without CO addition, a specific maximum H_2_ uptake rate of 71 mmol g_CDW_^−^^1^ h^−1^ was measured at 0.08 vvm, which represents an 61% increase compared to the cultivation with CO. This is in line with previous studies, where increased CO concentrations led to decreased H_2_ uptake rates and vastly prolonged lag phase durations during batch studies with *A. woodii* (wild type). These studies also revealed, that the reason for this inhibitory effect is most probably the CO sensitive hydrogenases of *A. woodii* [34, 37]. Under CO addition, reducing equivalents can still be generated via the CODH, making the activity of the HydABC less crucial for cell growth. Simultaneously, the inhibition of the HDCR becomes an important factor that hinders CO_2_ fixation to formate. Therefore, no formate was detected and no CO_2_ is taken up during the lag phase with 3% CO, followed by the first transition phase with increasing biomass formation. CO_2_ uptake was initiated after a certain biomass level was achieved in the stirred tank reactor after ∼ 80 h. Thus, the dissolved CO concentration (not measured) may have become low enough so that the HDCR was no longer inhibited, leading to acetate, d‐lactate and formate formation and accelerated growth. Simultaneously, also in line with prior studies, the addition of CO led to clearly increased maximum CDW concentrations and reduced acetate formation [34, 37]. This may be attributed to enhanced ATP generation through CODH‐derived Fd_red_ driving the Rnf complex, enabling greater anabolic activity and lowering the need for catabolic acetate production [36]. While initial CO concentrations in the liquid phase are likely similar across flow rates in the absence of cell activity, growth dynamics differ markedly. At 0.08 vvm with 3% CO, engineered *A. woodii* exhibited a prolonged lag phase and a lower growth rate (*µ* = 0.08 h^−^^1^), whereas at 0.25 vvm, growth began ∼48 h earlier and reached a significantly higher rate (*µ* = 0.18 h^−^^1^, Figure 3A). For comparison, the reference process without CO at 0.08 vvm reached *µ* = 0.13 h^−^^1^ (Figure 2A). This suggests that CO impairs early energy metabolism even before net uptake begins, delaying growth initiation. Once CO is consumed, gas transfer dynamics become more relevant: the higher flow rate at 0.25 vvm likely improves H_2_ availability and redox balance, supporting faster adaptation and biomass accumulation. In contrast, the extended lag phase at 0.08 vvm may reflect higher maintenance energy demands. Overall, these findings show that *A. woodii* can grow with 3% CO in stirred tank bioreactors if gas flow is sufficient—providing the basis for all subsequent experiments with lower CO concentrations at 0.25 vvm.

### 3.3 Gas Fermentations With Reduced CO Additions

**FIGURE 4:**
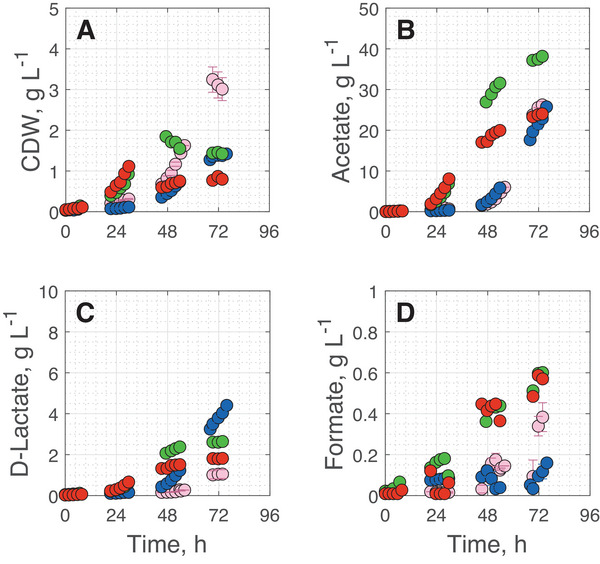
Cell dry weight (A), acetate (B), d‐lactate (C) and formate (D) concentrations during batch cultivation under autotrophic growth conditions of *A. woodii* Δ*pyrE* Δ*lctBCD* [pMTL83251_P*_lctA_* _NFP] in a stirred tank bioreactor with continuous gassing with 3.0% (v/v) CO in the inlet gas (rose), 1.0% (v/v) CO (red), 0.8% (v/v) CO (blue), and 0.6% (v/v) CO (green). (*T* = 30°C; pH  =  7.0; P V^−1^ = 6.06 W L^−1^, F_gas_  =  0.25 vvm). The error bars during cultivations with 0.8% CO indicate the minimum and maximum values of two independent batch processes under autotrophic growth conditions.

To take advantage of the increased biomass formation while reducing the inhibitory effects on the HDCR during cultivation with CO, smaller amounts of CO were added to the gas mixture. Building on the earlier growth initiation at 0.25 vvm with 3% CO, all further experiments were performed at this increased gas flow rate. Batch processes under autotrophic growth conditions were carried out at three different CO concentrations in the inlet gas. During these experiments, the gas mixture consisted of 70% H_2_ (v/v), 27 ‐ 29.4% CO_2_ (v/v), the respective amount of CO, and 0 ‐ 2.2% N_2_ (v/v) as a makeup gas. Tested CO concentrations were 0.6% (v/v), 0.8% (v/v), and 1.0% (v/v). Cell growth, acetate, formate, and d‐lactate concentrations are shown in Figure 4 and process performance data is presented in Table 3. To provide better context for the results, the cultivation data with 3% CO are displayed again here.

**TABLE 3:** Process performance data of autotrophic batch studies with *A. woodii* Δ*pyrE* Δ*lctBCD* [pMTL83251_P*_lctA_* _NFP]. Data represents maximum concentrations and production rates measured with 0.6%, 0.8%, and 1.0% CO in the inlet gas in fully controlled stirred tank reactor cultivations with continuous gassing (mean values of two independent processes). (*T*  =  30°C; pH  =  7.0; P V^−1^ = 6.06 W L^−1^, *F*_gas_  =  0.25 vvm) Values in brackets show the deviation from the autotrophic reference process without CO.

	0.6% CO		0.8% CO		1.0% CO	
CDW_max_, g L^−1^	1.85	(+ 8%)	1.37	(‐ 20%)	1.11	(−35%)
µ_max_, h^−1^	0.24	(+ 60%)	0.28	(+ 87%)	0.16	(+ 7%)
c_Acetate_, g L^−1^	38.17	(+ 7%)	22.66	(−37%)	24.03	(−33%)
q_Acetate_, g L^−1^ h^−1^	1.20	(−19%)	0.90	(−39%)	1.07	(−28%)
c_Lactate_, g L^−1^	2.64	(+ 41%)	4.00	(+ 114%)	1.82	(−3%)
q_Lactate_, g L^−1^ h^−1^	0.10	(+ 43%)	0.15	(+ 114%)	0.08	(+ 14%)
c_Formate_, g L^−1^	0.60	(−10%)	0.12	(−82%)	0.59	(−12%)
q_Formate_, g L^−1^ h^−1^	0.03	(−50%)	0.03	(−50%)	0.04	(−33%)
c_Acetate_ / c_Lactate_, g g^−1^	14.46	(−24%)	5.67	(−70%)	13.20	(−31%)

Contrary to the batch cultivations with 3% CO, growth of the lactate producing *A. woodii* cells was observed without a lag phase with 0.6% and 1.0% CO, whereas the cultivation of cells with 0.8% CO displayed a prolonged lag phase. Exponential cell growth was delayed by ∼ 22 h. Notably, maximum CDW concentrations were clearly reduced by 43% with 0.6% CO, by 58% with 0.8% CO, and by 66% with 1.0% CO compared to batch cultivations with 3% CO. As previously described, most experiments showed a significant decline in biomass concentration after reaching the maximum. However, even though the maximum CDW concentration during cultivations with 1.0% and less CO are not available, it seems unlikely that the CDW concentrations would reach comparable levels to the batch cultivation with 3% CO.

During all batch cultivations, acetate, D‐lactate and formate concentrations started to increase as soon as exponential biomass formation was initiated (Figure 3B–D). Highest final acetate concentrations of 38.17 g L^−1^ was observed with 0.6% CO. The highest final d‐lactate concentration of 4 g L^−1^ was measured with 0.8% CO in the artificial syngas (key process performance data are summarized in Table 2). Interestingly, formate concentrations were strongly reduced with 0.8% CO compared to the other batch cultivations throughout the whole process.

The rapidly initiating and strong exponential growth with 0.6% and 1.0% CO indicate no inhibition of the HDCR as observed before with 3% CO in the inlet gas. Therefore, the HDCR is unlikely responsible for the initial inhibitory effect on the lactate producing *A. woodii* at 0.8% CO in the inlet gas. Simultaneously, the high d‐lactate and low formate concentrations at 0.8% CO may indicate a shift in the availability and distribution of reduction equivalents in this batch cultivation. The reason is unknown. However, an overreduction of the cell due to a shift in metabolite and product spectrum may influence growth, leading to the observed prolonged lag phase [41]. Varying CO sensitivities of two different CODHs in *A. woodii*, namely the bifunctional CODH/ACS complex and a monofunctional CODH, may be an approach to explain this observation. However, the latter's existence has not yet been fully clarified for *A. woodii* [42]. Interestingly, this metabolic shift is consistent throughout the entire batch process. Therefore, elongation of the process time should result in increased d‐lactate concentrations.

### 3.4 Elongation of Gas Fermentations With 0.8% CO

**FIGURE 5:**
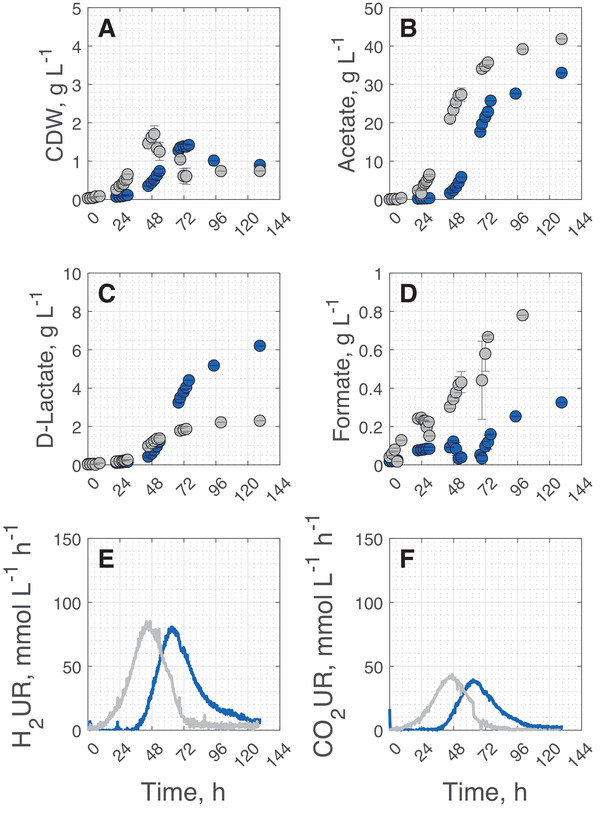
CDW (A), acetate (B), d‐lactate (C), formate (D) concentrations, and H_2_ (E) and CO_2_ uptake rates (F) during batch cultivation under autotrophic growth conditions of *A. woodii* Δ*pyrE* Δ*lctBCD* [pMTL83251_P*_lctA_* _NFP] with 0.8% (v/v) CO (blue) and without CO (gray) in the inlet gas (*T* = 30°C; pH  =  7.0; P V^−^** ^1^  = 6.06 W L*^−^* ^1^, *F*_gas_ = 0.25 vvm). The error bars indicate the minimum and maximum values of two independent batch processes under autotrophic growth conditions. We selected solely the representative H_2_ and CO_2_ uptake rates from one of the two independent processes for a more explicit representation.

Batch cultivations with 0.8% CO and without CO as reference were performed in stirred tank bioreactors with continuous gassing for 129 h. At this point, none of the experiments showed any further H_2_ or CO_2_ uptake. The results of the batch processes with engineered *A. woodii* are shown in Figure 5 and the key process performance data is presented in Table 4.

**TABLE 4:** Process performance data of autotrophic batch studies with *A. woodii* Δ*pyrE* Δ*lctBCD* [pMTL83251_P*_lctA_* _NFP] without and with 0.8% CO (v/v) in the inlet gas in a fully controlled stirred tank bioreactor. Indicated maximum concentrations and uptake rates represent the average values of two independent batch processes. The gas uptake rates represent the maxima of a single representative batch process (*T* = 30°C; pH = 7.0; P V^−1^ = 6.06 W L^−1^, *F*_gas_ = 0.25 vvm).

	H_2_ + CO_2_ and 0% CO	H_2_ + CO_2_ and 0.8% CO	Change with / without CO
CDW, g L^−1^	1.71	1.39	−19%
c_Acetate_, g L^−1^	42.69	33.01	−23%
c_Lactate_, g L^−1^	2.15	6.21	+ 189%
c_Formate_, g L^−1^	0.88	0.33	−63%
c_Acetate_ / c_Lactate_, g g^−1^	19.86	5.32	−73%
H_2_UR, mmol L^−1^ h^−1^	85.00	80.72	−5%
CO_2_UR, mmol L^−1^ h^−1^	43.56	39.50	−9%
H_2_UR / CO_2_UR, mol mol^−1^	1.95	2.04	+ 5%

Even though the maximum CDW concentrations were reduced by 20% during batch cultivations with 0.8% CO compared to the reference without CO addition, the final biomass concentrations approached similar values (Figure 4A). As shown before (Figure 1), in contrast to the reference batch process without CO, H_2_ and CO_2_ uptake rates were negligible with 0.8% CO until exponential growth was initiated. The H_2_ and CO_2_ uptake rates peaked, as soon as the maximum CDW concentrations were reached (Figure 3E,F). With 0.8% CO, even though the maximum CDW concentrations were reduced by 19%, the maximum H_2_ and CO_2_ uptake rates were only reduced by 5%, and 9%, respectively. After reaching their maxima, the H_2_ and CO_2_ uptake rates decreased rapidly in the reference process without CO, while the decline was less pronounced with 0.8% CO in the inlet gas.

Batch process elongation resulted in increased final product concentrations (acetate, d‐lactate, and formate) with and without CO addition (Figure 3B,C). With 0.8% CO, 6.21 g L^−1^ d‐lactate were accumulated until a process time of 129 h (5.4 days) which represents an increase by 189% compared to the reference process without CO (key process performance data are compared in Table 4). A further extension of the process time would most probably result in even higher d‐lactate concentrations, since concentrations continued to increase until a process time of 129 h.

As already observed before, although significantly less biomass was produced with 0.8% CO compared to 0% CO, the reduction in the H_2_ uptake rate was less pronounced. With 0% CO, the maximum biomass specific H_2_ uptake rate was 49.7 mmol g_CDW_^−1^ h^−1^. With 0.8% CO, the maximum specific H_2_ uptake rate increased to 58.1 mmol g_CDW_^−1^ h^−1^. The enhanced maximum specific H_2_ uptake rate indicates increased HydABC activity compared to cultivations without CO addition. At higher HydABC activity, more NADH and Fd_red_ can be formed. Thus, more NADH is available to produce D‐lactate with LDHD. In addition, the formation of pyruvate from acetyl‐coenzyme A needs CO_2_. The increased and continuing formation of D‐lactate from pyruvate might therefore be the reason for the extended H_2_ and CO_2_ uptake period with 0.8% CO.

Despite the substantial improvement of the final d‐lactate concentration by adding 0.8% CO (from 2.51 to 6.21 g L^−1^), the final D‐lactate concentration is significantly lower compared to *A. woodii* expressing the lactate dehydrogenase of *L. mesenteroides* under the control of a lactose inducible promotor (P*_bgaL_*) in a stirred tank bioreactor with continuous gassing of CO_2_ and H_2_ without CO (8.1 g L^−1^ D‐lactate) [4]. The P*_bgaL_* promoter reduced the exponential growth rate of *A. woodii* (*µ*_max_ = 0.08 h^−1^) already prior to and strongly after induction despite doubled yeast extract concentration in the medium (6 g L^−1^ compared to 3 g L^−1^) most probably due to metabolic burden by higher expression of the lactate dehydrogenase. In our study, the introduction of an alternative promoter for lactate production, P*_lctA_*, allowed improved growth of the lactate producing *A. woodii*, resulting in exponential growth rates of *µ*_max_ = 0.15 h^−1^ (without CO addition) similar to batch processes under autotrophic growth conditions with *A. woodii* wild type, e.g., *µ*_max_ = 0.13 h^−1^ [34].

## 4 Conclusion

Our studies applying a fully controlled stirred tank bioreactor with continuous gassing demonstrated that even minimal amounts of CO in the artificial syngas significantly affect growth, and the formation of products in batch cultivations under autotrophic growth conditions of engineered *A. woodii* overexpressing the D‐lactate dehydrogenase (LDHD) gene (*ldhD*) from *L. mesenteroides* under control of the lactate inducible promoter P*_lctA_*. Surprisingly, batch process dynamics and product concentrations are non‐linearly dependent on the initial CO concentrations (0.6%–6.0% CO). For example, high maximum CDW concentrations were observed with higher CO concentrations (3.0% CO), and highest d‐lactate accumulation was solely possible with 0.8% CO. The high sensitivity of *A. woodii* to varying CO concentrations in the artificial syngas needs further studies, for example, on the CO‐sensitivity of the enzymes involved in the WLP, and the carbon flux distributions altered by CO. In addition, the measurement of the CO concentrations in the liquid phase [48] would be beneficial to identify threshold CO concentrations causing metabolic shifts in *A. woodii*.

## Author Contributions

Conceptualization: Anna Stock and Dirk Weuster‐Botz. Methodology and investigation: Anna Stock and Inka Sotzeck (syngas fermentation), Kira Baur and Frank Bengelsdorf (metabolic engineering). Data discussion and analysis: Anna Stock, Inka Sotzeck, Kira Baur, Frank Bengelsdorf, and Dirk Weuster‐Botz. Writing – original draft preparation: Anna Stock and Kira Baur (metabolic engineering). Writing – review and editing: Dirk Weuster‐Botz, and Frank Bengelsdorf. Visualization: Anna Stock. Supervision, project administration and funding acquisition: Dirk Weuster‐Botz, and Frank Bengelsdorf. All authors have read and agreed to the published version of the manuscript.

## Conflicts of Interest

The authors declare no conflicts of interest.

## Supporting information




**Supplementary File 1**: elsc70072‐sup‐0001‐SuppMat.docx

## Data Availability

The data that support the findings of this study are available from the corresponding author upon reasonable request.
