# Supplementary material for: Careful CO Addition Enhances Autotrophic d‐Lactate Formation With Engineered Acetobacterium woodii
Source: Eng Life Sci. 2026 Feb 17;26(2):e70072. doi: 10.1002/elsc.70072 (PMC12910521; doi:10.1002/elsc.70072)
Supplement: Supplementary file 1 — elsc70072‐sup‐0001‐SuppMat.docx [file ELSC-26-e70072-s001.docx]

**Supplementary material**

**Bioreactor media compositions**

Table S1: Composition of the mod. Novak medium (Novak et al., 2021) for bioreactor cultivations

| **Component** | **Concentration, g L^-1^** |
| --- | --- |
| Yeast Extract | 3.0 |
| Uracil | 0.02 |
| NaCl | 3.47 |
| NH_4_Cl | 1.00 |
| KH_2_PO_4_ | 0.33 |
| K_2_HPO_4_ | 0.45 |
| **Component** | **Concentration, mL L^-1^** |
| Trace element solution | 40.0 |
| MgSO_4_ *_·_* 7 H_2_O solution (165 g L*^−^*^1^) | 0.6 |
| Vitamin solution | 20.0 |
| L-Cysteine-HCl solution (50 g L*^−^*^1^) | 10.0 |

Table S2: Vitamin solution concentrations (DSMZ 141)

| **Component** | **Concentration, mg L^-1^** |
| --- | --- |
| D-Ca-Pantothenate | 50 |
| Pyridoxine-HCl | 10 |
| Thiamine-HCl | 5 |
| Riboflavin | 5 |
| Nicotinic acid | 5 |
| α-aminobenzoic acid | 5 |
| Lipoic acid | 5 |
| Biotin (B7) | 2 |
| Folic acid (B9, B11) | 2 |
| Cyanocobalamine (B12) | 0.1 |

Table S3: Trace element solution concentrations (mod. DSMZ 151)

| **Component** | **Concentration, g L^-1^** |
| --- | --- |
| MgSO_4_ *·* 7 H_2_O | 3 |
| Nitrilotriacetic acid | 1.5 |
| FeSO_4_ *·* 7 H_2_O | 1.35 |
| MnSO_4_ *·* H_2_O | 0.5 |
| NaCl | 1 |
| CoCl_2_ *·* 6 H_2_O | 0.152 |
| CaCl_2_ *·* 2 H_2_O | 0.1 |
| ZnSO_4_ *·* 7 H_2_O | 0.18 |
| CuSO_4_ *·* 5 H_2_O | 0.01 |
| KAl(SO_4_)_2_ *·* 12 H_2_O | 0.02 |
| H3BO3 | 0.01 |
| Na_2_MoO_4_ *·* 2 H_2_O | 0.01 |
| NiSO_4_ *·* 6 H_2_O | 0.033 |
| **Component** | **Concentration, mg L^-1^** |
| Na_2_SeO_3_ *·* 5 H_2_O | 0.3 |
| Na_2_WO_4_ *·* 2 H_2_O | 0.4 |

**References**

Novak, K., Neuendorf, C. S., Kofler, I., Kieberger, N., Klamt, S., & Pflügl, S. (2021). Blending industrial blast furnace gas with H2 enables Acetobacterium woodii to efficiently co-utilize CO, CO2 and H2. *Bioresource Technology*, *323*, 124573. https://doi.org/10.1016/j.biortech.2020.124573
